# Development of Tofacitinib Loaded pH-Responsive Chitosan/Mucin Based Hydrogel Microparticles: In-Vitro Characterization and Toxicological Screening

**DOI:** 10.3390/gels9030187

**Published:** 2023-02-28

**Authors:** Rania T. Malatani, Sana Bilal, Asif Mahmood, Rai Muhammad Sarfraz, Nadiah Zafar, Hira Ijaz, Umaira Rehman, Shehla Akbar, Hala M. Alkhalidi, Heba A. Gad

**Affiliations:** 1 Department of Pharmacy Practice, Faculty of Pharmacy, King Abdulaziz University, Jeddah 21589, Saudi Arabia; 2 Faculty of Pharmacy, The University of Lahore, Lahore 54000, Pakistan; 3 Department of Pharmacy, University of Chakwal, Chakwal 48800, Pakistan; 4 College of Pharmacy, University of Sargodha, Sargodha 40100, Pakistan; 5 Department of Pharmaceutics, Faculty of Pharmacy, Universiti Teknologi MARA, Puncak Alam Campus, Bandar, PuncakAlam 42300, Malaysia; 6 Department of Pharmaceutical Sciences, Pak-Austria Fachhochschule: Institute of Applied Sciences and Technology, Mang, Khanpur Road, Haripur 22620, Pakistan; 7 Department of Pharmacognosy, Faculty of Pharmacy, The Islamia University of Bahawalpur, Bahawalpur 63100, Pakistan; 8 Department of Pharmaceutics and Industrial Pharmacy, Faculty of Pharmacy, Ain Shams University, Cairo 11566, Egypt; 9 Department of Pharmaceutical Sciences, Pharmacy Program, Batterjee Medical College, P.O. Box 6231, Jeddah 21442, Saudi Arabia

**Keywords:** tofacitinib, mucin, chitosan, arthritis, permeation

## Abstract

Tofacitinib is an antirheumatic drug characterized by a short half-life and poor permeability, which necessitates the development of sustained release formulation with enhanced permeability potential. To achieve this goal, the free radical polymerization technique was employed to develop mucin/chitosan copolymer methacrylic acid (MU-CHI-Co-Poly (MAA))-based hydrogel microparticles. The developed hydrogel microparticles were characterized for EDX, FTIR, DSC, TGA, X-ray diffraction, SEM, drug loading; equilibrium swelling (%), in vitro drug release, sol–gel (%) studies, size and zeta potential, permeation, anti-arthritic activities, and acute oral toxicity studies. FTIR studies revealed the incorporation of the ingredients into the polymeric network, while EDX studies depicted the successful loading of tofacitinib into the network. The thermal analysis confirmed the heat stability of the system. SEM analysis displayed the porous structure of the hydrogels. Gel fraction showed an increasing tendency (74–98%) upon increasing the concentrations of the formulation ingredients. Formulations coated with Eudragit (2% *w*/*w*) and sodium lauryl sulfate (1% *w*/*v*) showed increased permeability. The formulations equilibrium swelling (%) increased (78–93%) at pH 7.4. Maximum drug loading and release (%) of (55.62–80.52%) and (78.02–90.56%), respectively, were noticed at pH 7.4, where the developed microparticles followed zero-order kinetics with case II transport. Anti-inflammatory studies revealed a significant dose-dependent decrease in paw edema in the rats. Oral toxicity studies confirmed the biocompatibility and non-toxicity of the formulated network. Thus, the developed pH-responsive hydrogel microparticles seem to have the potential to enhance permeability and control the delivery of tofacitinib for the management of rheumatoid arthritis.

## 1. Introduction

The American Organization of Rheumatology recommends using disease-modifying antirheumatic drugs (DMARDs) for moderate-to-severe disease response. They are used as monotherapy or in combination for the early stages of rheumatoid arthritis. To control the progression of the disease, the use of biological DMARDS and anti-TNFα either alone or together with methotrexate (MTX) is suggested [1].

Tofacitinib is an emerging DMARD used for the treatment of moderate to severe rheumatoid arthritis, active psoriatic arthritis, as well as ulcerative colitis [2]. It acts by inhibiting Janus kinases [3], where it is considered a selective Janus kinase inhibitor (JAK) with preferential repression/inhibition of JAK3 and JAK1 and to some extent, JAK2 [4]. According to biopharmaceutics classification system (BCS), it belongs to Class III, i.e., highly soluble with low permeability [5]. Tofacitinib is commercially available in dosage forms such as immediate release (IR) tablets, extended release (XR) tablets, and as oral solution preparations. Tofacitinib, being well absorbed by gastrointestinal tract (GIT) after oral administration, reaches its peak plasma concentration (C_max_) after 0.5–1 h. It has a short half-life of about 3.2 h. [5] and is soluble in water, ethanol, and dimethyl sulfoxide (DMSO). Adverse effects of tofacitinib include increased levels of alanine transaminase (ALT) and aspartate aminotransferase (AST), nasopharyngitis, dyspnea [6], anemia, hyperlipidemia, and increased risk of cardiovascular diseases such as leukopenia [7] and thromboembolism.

Rheumatoid arthritis (RA) is an autoimmune disease of the synovial joints [8]. The etiology of the disease is still unknown, although genes (HLA) and environmental factors (smoking) are identified as playing a significant role in prognosis of RA [9]. Studies have proven that the disease nearly affects 0.5 to 1% of the total world population, particularly females and the elderly population [10]. It is characterized mainly by synovial inflammation and joint disfigurement [8]. Its pathophysiology progresses with the onset of nonspecific inflammation followed by amplified number of T cells, later leading to chronic stage with activation of cytokines, i.e., IL-1, TNF-alpha, and IL-6, thus causing inflammation and tissue damage [11,12].

Mucin is a high molecular weight glycoconjugate with a polypeptide backbone, and oligosaccharide side chains attached to this backbone via covalent bonding of O–glycosidic linkages [13,14]. It is highly abundant in animal and human tissues and is present in all segments of wet epithelium, including the nasal, oral, gastric tract, ophthalmic system, salivary glands, female reproductive system, and respiratory tract [15,16]. It offers excellent biocompatibility, least toxicity, and is easily biodegradable when used for mucoadhesive systems [15]. It possesses hydrating, lubricating, and protective potentials against most disease-causing microbes [17,18]. Moreover, it has an excellent retention time, and a nonimmunogenic and nontoxic nature that makes it an ideal polymeric material for developing drug delivery systems in the pharmaceutical sector [19]. In the literature, hydrogels containing mucin crosslinked with methacrylic acid (MAA) using a covalent crosslinking technique have been developed and investigated for drug delivery purposes. These offer controlled release formulations of both water (H_2_O) soluble and insoluble drugs when used in combination with chitosan, lectin and polyethylene glycol, alginate, and gelatin [19].

Chitosan (CS), β–1,4–N–acetyl–D–glucosamine, has structural characteristics similar to mucopolysaccharides/glycosaminoglycans. It is a naturally occurring cationic polymer obtained from chitin by deacetylation. The shells of marine arthropods, i.e., lobsters, crabs and shrimps, are the current sources of chitin [20]. At acidic pH, it carries a positive charge that allows the formation of electrostatic complexes or multiple networks with other anionic polymers (natural or synthetic) [21]. Moreover, the presence of hydroxyl (OH) and amine (NH_2_) groups on chitosan contribute to hydrogen bonding [22].

Chitosan-based hydrogels can be prepared either directly from chitosan or in combination with other polymers. The surface charge on these chitosan hydrogels changes with pH, making them pH-responsive. However, being highly soluble in low/acidic pH and low heat stability, it exhibits limited mechanical properties and uncontrolled drug release [23,24,25]. For such hydrogel systems to be potential drug delivery carriers, chemical modification is carried out in amino (NH_2_) and hydroxyl (OH) groups. Crosslinking agents including glutaraldehyde (GLA) [26], formaldehyde [27], ethylene glycol diglycidyl ether (EGDGE) [28] and N, N’-methylenebisacrylamide (MBA), etc., are being tried in the literature [25].

MBA is a crosslinker that has two identical double bonds. MBA as a crosslinking agent results in highly resilient hydrogels [29,30]. MAA is a monomer with aqueous solubility, heat sensitivity and biocompatibility [31]. It contains carboxylic acid (-COOH) groups and carbon–carbon double bonds (C=C). Its property to copolymerize and market availability makes it a candidate of choice for the development of hydrogels [32]. In response to pH change, ionization of carboxylic acid (-COOH) groups occurs, thereby affording them a pH-responsive character. The addition of the acid/monomer in hydrogels imparts pH sensitivity and changes their swelling properties. Ammonium persulfate (APS) is an organic compound used as an initiator. It has two positively charged ammonium and one negatively charged peroxydisulfate ion [33,34]. It is a chemical substance with high aqueous solubility, lower pH and viscosity, least toxicity, and cost-effectiveness [35].

Hydrogels are three-dimensional (3D) hydrophilic networks with the ability to incorporate large amounts of physiological fluids [36,37]. They emerged as valuable drug delivery materials due to elasticity [38], biocompatibility [39], permeability [40], nonimmunogenic nature, and least toxicity [39]. Due to their ability to change their properties in response to external environmental factors such as pH, temperature, light, and enzymes [36,40], they are being named smart/stimulus-responsive hydrogels [41]. For targeted drug delivery, pH-responsive hydrogels are ideal candidates. The low stomach pH (<3) and the higher intestine pH (>6) are enough to initiate pH-dependent swelling based on environmental pH change [32].

Lately, natural polymers such as chitosan, pectin, collagen, gelatin, dextran, albumin, alginate, and cellulose, etc., are being used for development of such stimuli-responsive hydrogels [40,42,43]. Different techniques have been presented in the literature for the preparation of hydrogels, including bulk polymerization, solution polymerization, suspension polymerization or inverse-suspension polymerization, free radical polymerization, grafting, polymerization using irradiation and crosslinking (chemical and physical), etc., [44,45].

This study aimed to develop and optimize tofacitinib containing pH-responsive polymeric microparticles for sustained tofacitinib delivery with promising permeability enhancement potentials. Hydrogel microparticles were developed using different ratios of mucin, chitosan, MAA, and MBA. Developed hydrogels were characterized using FTIR, DSC, XRD, EDX, TGA, SEM, zeta size, zeta potential, swelling (%), release (%), and sol–gel studies. Biocompatibility was evaluated by performing acute oral toxicity studies.

## 2. Results and Discussion

### 2.1. Physical Appearance

**Figure 1:**
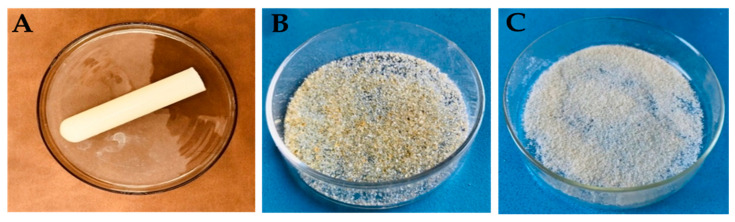
Physical appearance of MU-CHI-Co-Poly (MAA) hydrogel rod (**A**), dried unloaded hydrogel microparticles (**B**), and coated hydrogel microparticles (**C**).

Hydrogel microparticles were formulated using variable quantities of polymers (chitosan and mucin), crosslinker (MBA), and monomer (MAA). APS was used as an initiator for radical polymerization. Developed hydrogels were soft, elastic, and slightly yellowish in appearance. A total of twelve formulations were prepared. All the synthesized hydrogels presented excellent strength. The appearance of the developed hydrogel (A), dried unloaded hydrogel microparticles (B), and coated MU-CHI-Co-Poly (MAA) polymeric microparticles (C) is presented in Figure 1.

Results revealed the enhanced effect of different ingredients on the appearance of the developed formulations. When the amount of chitosan (0.2–0.4 g) was increased, milky-colored hydrogels with soft rubbery texture were acquired. Moreover, with the increase in quantities of mucin (0.1–0.3 g) while all the other ingredients being constant (chitosan, MAA, MBA), hydrogels with light yellowish color were attained with pronounced integrity. Furthermore, by increasing the amount of MBA (0.3–0.7 g), hydrogels with rigid texture were obtained. Likewise, there was no apparent change in color.

### 2.2. Elemental Analysis

**Figure 2:**
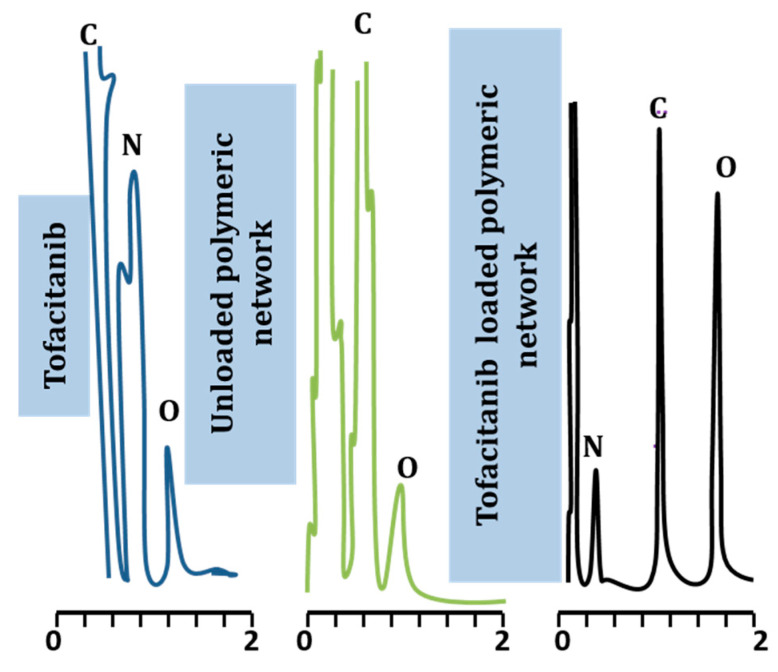
EDX spectra of Tofacitinib, unloaded microparticles, tofacitinib-loaded network, where C, N, and O represent carbon, nitrogen, and oxygen, respectively.

EDX analysis was carried out to confirm the elemental composition of ingredients utilized for development of the polymeric network. Furthermore, it also evaluates the % atomic weight of each element present in the sample. The EDX spectrum of tofacitinib, unloaded hydrogel microparticles, and tofacitinib-loaded hydrogel microparticles was recorded. The EDX spectrum of tofacitinib, as shown in Figure 2, presented carbon (41.25%), nitrogen (33.72%), and oxygen (25.03%). These elements were also present in the chemical structure of the drug. The EDX spectrum of unloaded hydrogel microparticles revealed the presence of carbon (59.42%) and oxygen (39.15%). The spectrum of tofacitinib-loaded hydrogel microparticles contained oxygen (39.24%), carbon (34.63%), and nitrogen (12.42%) as shown in Table 1. Moreover, nitrogen is a characteristic part of tofacitinib that was detected in the drug-loaded hydrogels. Successful tofacitinib loading was validated due to presence of nitrogen contents within the hydrogel microparticles. EDX spectrum proved presence of the drug within the network from the existence of a nitrogen peak in the case of the drug-loaded hydrogel microparticles.

### 2.3. Fourier Transforms Infrared Spectroscopy

**Figure 3:**
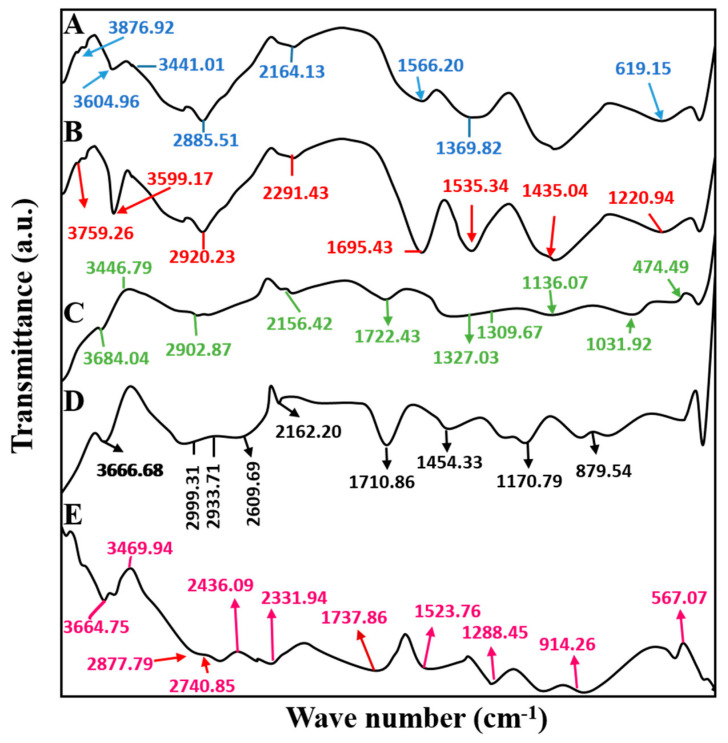
FTIR Spectra of (A) chitosan, (B) mucin, (C) tofacitinib, (D) unloaded microparticles, (E) drug-loaded MU-CHI-Co-Poly (MAA) hydrogel microparticles.

IR spectrum of pure CS was recorded to find out presence of functional groups in the structural composition of the polymer. FTIR analysis was carried out at a wavelength range of 4000–400 cm^−1^. Chitosan spectrum presented a band at 3876 cm^−1^ due to stretching vibrations of O–H group. The bands between 3508 cm^−1^ to 3441 cm^−1^ were attributed to bending vibrations of the amine group (N–H). The two distinct bands observed at 2885 cm^−1^ and 2164 cm^−1^ were related to the oscillation of aliphatic groups (-CH_2_ and -CH_3_). The absorption band at 1566 cm ^−1^ can be assigned to the amide II band due to the bending of the -NH_2_ group. It reflects that chitosan was partially obtained by deacetylation of chitin. The prominent band at 1359 cm^−1^ represented the stretching of C–C, which was due to the presence of glucosamine groups in chitosan. Meanwhile, the absorption band at 619 cm^−1^ corresponds to stretching vibrations of O=C–N group. Results are shown in Figure 3A.

The FTIR spectrum of mucin presented in Figure 3B showed characteristics band at 3759 cm^−1^ corresponding to stretching vibrations of the O–H group. Sharp and fewer intensive bands observed from 3759–3599 cm^−1^ were due to O–H/N–H oscillations of secondary amines, alcohol, and amides. The band obtained at 2920 cm^−1^ was because of the bending of the aromatic group (C–H). Furthermore, CN stretching vibration at 2291 cm^−1^ and C=O bending at 1695 cm^−1^ were seen. In addition, absorption bands at 1535 cm^−1^, 1435 cm^−1^, and 1220 cm^−1^ were related to the bending of amide II, C–H, and amide III, respectively. The band at 1060 cm^−1^ corresponded to the vibration of carbonyl group (C=O). Lastly, any bands observed below 800 cm^−1^ can be assigned to out-of-plane bending of the C–H group.

IR spectrum of tofacitinib presented in Figure 3C showed noticeable bands at 3684 cm^−1^ and 3446 cm^−1^ due to stretching of the O–H group. Characteristic bands at 2902 cm^−1^ and 2156 cm^−1^ corresponded to N–H and C–C stretching vibrations. Furthermore, symmetric stretching vibrations of the carbonyl group (C=O) at 1722 cm^−1^ and oscillations of the S=O group were seen at 1327 cm^−1^ and 1309 cm^−1^. Typical bands were noticed at 1136 cm^−1^ due to bending vibrations of the aliphatic group (C–O), C–N vibration at 1031 cm^−1^, and silicone rocking vibration at 474 cm^−1^ being observed, respectively.

The FTIR spectrum used to analyze unloaded microparticles is presented in Figure 3D. The absorption band at 3666 cm^−1^ indicated O–H stretching vibrations. The band that appeared at 2999 cm^−1^ showed bending of =C–H, while the corresponding band at 2933 cm^−1^ was related to the stretching of alkanes (C–H). A wide band at 2609 cm^−1^ represents stretching vibration of N–H and the presence of a band at 2162 cm^−1^ was substituted to CN oscillations. The symmetric stretching vibration of the carboxylic group (C=O) was observed at 1710 cm^−1^. The presence of wave no. at 1454 cm^−1^ was stretching of the C=C group. The appearance of bands at 1170 cm^−1^, 879 cm^−1^, and 831 cm^−1^ corresponded to vibrations of alkyl amines’ in-plane bending of the C–O group and out-of-plane bending of the aromatic group (C–H).

IR analysis performed for loaded MU-CHI-Co-Poly (MAA) hydrogel microparticles is presented in Figure 3E. Sharp bands of mucin obtained at 3759–3599 cm^−1^ due to sharp O–H stretching vibrations were less intensified and shifted to 3664–3593 cm^−1^. Bands observed in unloaded microparticles at 2999–2933 cm^−1^ were shifted to 2877–2740 cm^−1^ due to C–H stretching of the methyl group. The bands that appeared at 2436 cm^−1^ and 2331 cm^−1^ corresponding to C–C oscillations. A prominent wide band at 1737 cm^−1^ was assigned to the stretching of the carbonyl group (C=O). The absorption band seen in chitosan at 1566 cm^−1^ due to bending vibration of amide groups was shifted to a new position at 1523 cm^−1^. A sharp band at 1288 cm^−1^ was related to the symmetric stretching of primary amines (-NH_3_). Out-of-plane bending of the C–H group was noticed at 914 cm^−1^, whereas the band at 567 cm^−1^ was assigned to the rocking vibration of the C–S group. Band shifting, vanishing of any spectral band or emergence of new bands at any wavenumber confirms formation of the new polymeric complex.

### 2.4. Differential Scanning Calorimeter and Thermogravimetric Analysis

DSC analysis was conducted for hydrogels to understand thermal stability and phase transition behavior of the samples. TGA studies were conducted against rising temperatures to observe weight loss and weight changes against increasing temperature. DSC and TGA thermograms of pure drug tofacitinib, polymers (chitosan, mucin), and loaded and unloaded hydrogel microparticles were recorded at a temperature range of 0–500 °C.

**Figure 4:**
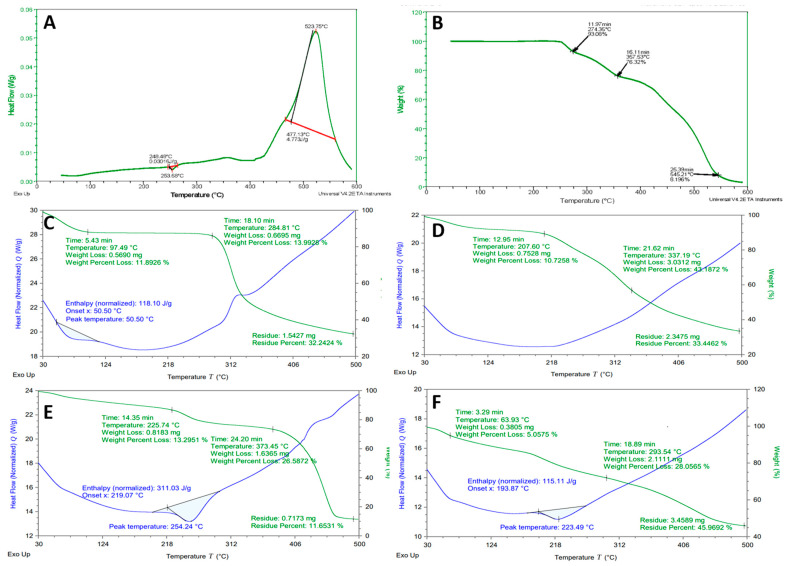
DSC and TGA thermograms: (**A**) DSC of Tofacitinib, (**B**) TGA of Tofacitinib, (**C**) chitosan, (**D**) mucin, (**E**) unloaded microparticles, (**F**) drug-loaded MU-CHI-Co-Poly (MAA) hydrogel microparticles. The blue color shows DSC and the green color shows TGA in (**C**–**F**).

DSC thermogram of pure drug tofacitinib is shown in Figure 4A. It indicates phase transition at 253.68 °C from solid to liquid with an enthalpy variation of 0.03016 J/g. This phase transition corresponded to the melting point of the drug. A prominent exothermic peak was noted at 523.75 °C, reflecting the complete combustion of the drug. TGA thermogram of pure drug tofacitinib is presented in Figure 4B. Drug decomposition occurred in three stages. Step I showed an initial weight loss of about 7% due to the loss of water content at 274.35 °C. Second degradation was seen at 357.53 °C with 23.68% weight loss. A maximum mass loss of 91.9% of the drug was observed at 545.21 °C.

DSC and TGA thermograms of pure chitosan are shown in Figure 4C. DSC thermogram exhibited an endothermic peak at around 73 °C indicating the evaporation of absorbed moisture with enthalpy change of 118.10 J/g. The second exothermic peak was observed around 312 °C, which is associated with the degradation of polymer and decomposition of acylated and deacylated units of chitosan. TGA thermogram of chitosan exhibited percentage weight loss in three stages. In the first stage, 11.89% weight loss was seen due to moisture loss at 97.49 °C. Stage two revealed a mass loss of about 13.99% at 284.81 °C due to the breakage of glycosidic linkages. A further rise in temperature above 300 °C led to major decomposition of the polymer.

DSC and TGA thermograms of pure mucin are presented in Figure 4D. In DSC thermogram, two endothermic peaks were observed. The first peak suggested the melting point of the mucin at 92 °C. The second peak depicted any retaining moisture loss at 218 °C. These peaks were followed by an exothermic peak above 300 °C because of the complete combustion of the polymer. In the TGA thermogram of mucin, the initial decrease in weight of 10.72% was noted at 207.60 °C due to the evaporation of water contents. Upon further heating up to 337.19 °C, the sample lost its weight by about 43.18%. Major degradation events occur at temperatures above 350 °C and have a residual mass of about 33.44% due to incomplete pyrolysis.

DSC and TGA thermograms of prepared unloaded microparticles are shown in Figure 4E. In the DSC thermogram, decomposition of the formulation occurred in two steps. The first endothermic peak was observed at 254.24 °C with an enthalpy variation of 311.03 J/g due to loss of moisture content. The second exothermic peak was seen at around 470 °C, which showed complete combustion of the polymeric network. The thermogram of unloaded developed microparticles presented an initial mass loss of 13.29% at 225.74 °C. At higher temperatures of 373.45 °C mass of the product lost was 26.58%. A further increase in temperature above 400 °C led to the decomposition of the microparticles, but even at elevated temperature, 11.65% of mass remained intact, thereby ensuring the stability of the formulated hydrogel microparticles.

DSC and TGA thermogram of drug-loaded MU-CHI-Co-Poly (MAA) hydrogel microparticles are depicted in Figure 4F. The DSC thermogram of the formulation indicated heat absorption at 223.49 °C, reflecting a lack of moisture content. In the TGA thermogram, initial mass loss was at 63.93 °C due to the evaporation of water. Heating at elevated temperature, i.e., 293.54 °C, resulted in weight loss of 28.05%. It was observed that even at a temperature higher than 400 °C, 45% mass of the polymeric network remained intact. Hence, the developed network was more stable when compared with thermal profile of formulation ingredients and it also ensures the stability of the incorporated drug. Results of 10% weight loss and char yield at 500 °C are presented in Table S1 as a supplementary file.

### 2.5. X-ray Diffraction Analysis

XRD studies of the drug (tofacitinib) and the MU-CHI-Co-Poly (MAA) unloaded and loaded polymeric network were carried out to comparatively analyze their nature, i.e., amorphous or crystalline. Each XRD diffractogram presented distinctive peaks and patterns when subjected to scanning in a range of 5–80°. Samples with crystalline nature exhibited sharp peaks thus reflecting poor dissolution rate and solubility, whereas fused peaks confirmed the amorphous nature of the material with enhanced solubility and good dissolution profile.

**Figure 5:**
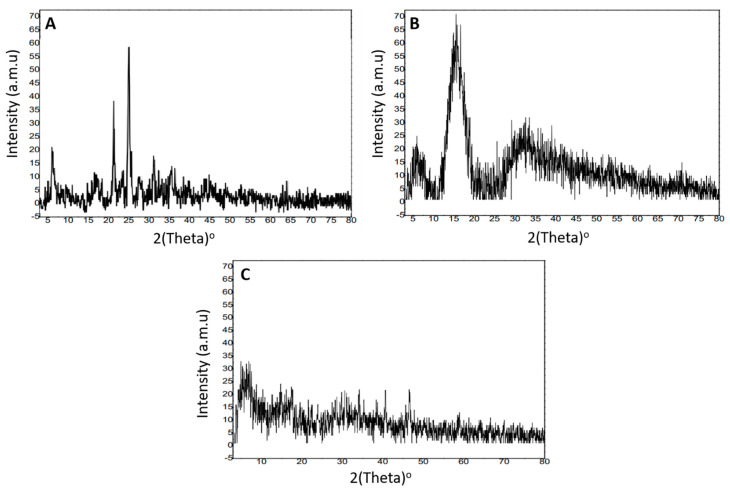
X-ray diffractogram of (**A**) tofacitinib, (**B**) unloaded microparticles, (**C**) drug-loaded MU-CHI-Co-poly (MAA) hydrogel microparticles.

XRD diffractogram of tofacitinib is presented in Figure 5A. It exhibited prominent and sharp peaks at an angle of 2*θ* = 7.54°, 21.37°, and 25.64°, confirming the crystalline nature of the drug. Moreover, fewer non-evident peaks were also observed at 31.47° and 35.95°.

XRD diffractogram of MU-CHI-Co-Poly (MAA)-unloaded microparticles is shown in Figure 5B. It presented a broad peak at 2*θ* = 15.97° whereas the majority of peaks were fused, indicating the successful crosslinking of the ingredients.

The XRD diffractogram of MU-CHI-Co-Poly (MAA) hydrogel microparticles is shown in Figure 5C. It presented no characteristic peaks, which were evident in the diffractogram of tofacitinib at 2*θ* = 7.54°, 21.37°, 25.64°,31.47°, and 35.95°. This concluded the change in crystallinity of the drug into an amorphous form and the incorporation of tofacitinib within the microparticles. Decrease in intensity of drug peaks indicated that the dissolution profile of the incorporated drug increased.

### 2.6. Scanning Electron Microscopy

SEM analysis was conducted to investigate the surface morphology of developed MU-CHI-Co-Poly (MAA) hydrogel microparticles. The study was carried out at various magnification powers, i.e., 100×, 250×, 500×, 1000×, 2500×, and 5000×, to check the presence of cracks, pores, surface appearance, and channels, if any.

**Figure 6:**
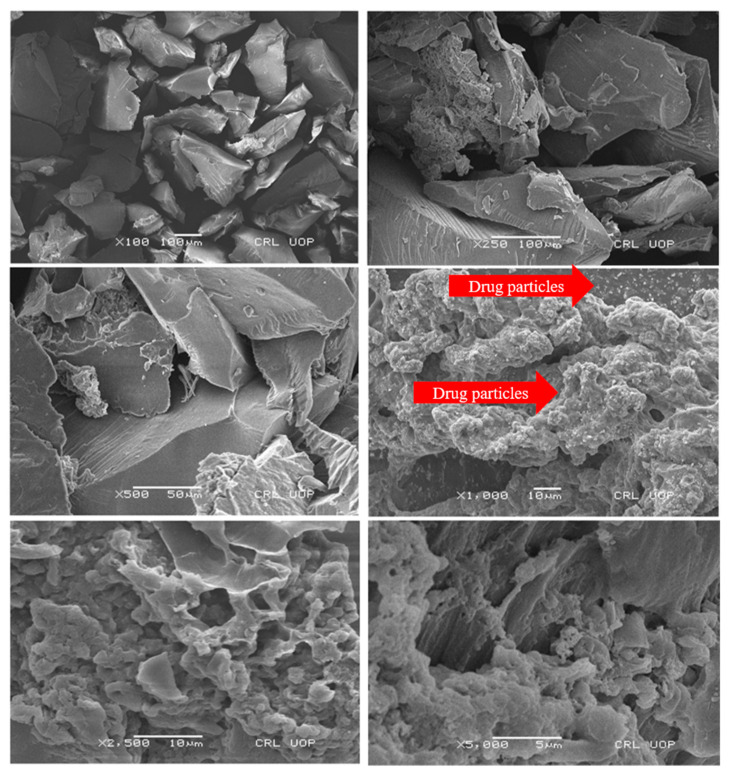
Photomicrographs of MU-CHI-Co-Poly (MAA) hydrogel microparticles.

The formulated MU-CHI-Co-poly (MAA) polymeric matrix showed a glossy, highly porous, slightly cracked and moderately wavy structure. Slight cracks and waves can be associated with overlapping polymeric chains during drying. Micrographs also confirmed the presence of porous surfaces that plays an important role in drug loading, release of therapeutic agents, water retention, and uptake of physiological media, thereby promoting the swelling of the microparticles. Moreover, the presence of whitish spots confirms the loading of tofacitinib. Results are presented in Figure 6.

### 2.7. Drug Loading (%)

**Figure 7:**
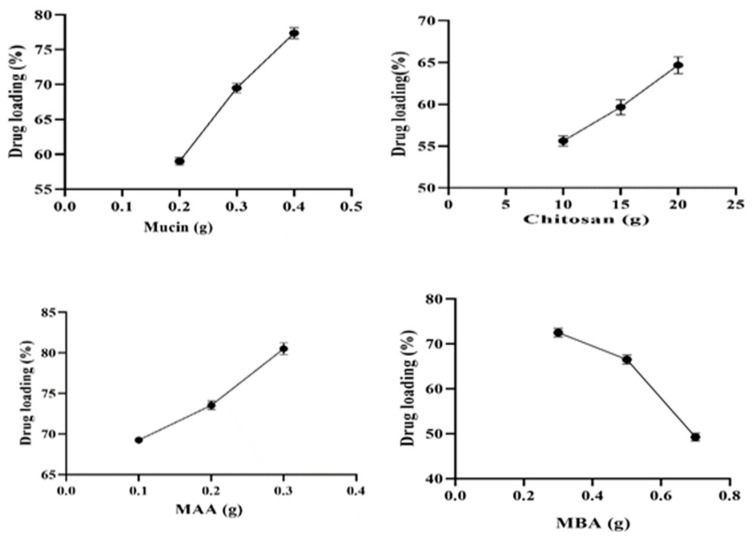
Effect of mucin, chitosan, MAA, and MBA on Tofacitinib loading.

The extent of tofacitinib loading (%) in all developed formulations (MCT1–MCT12) was observed by varying the concentration of the ingredients, i.e., mucin, chitosan, MAA, and MBA. All the results are displayed in Figure 7.

Higher loading of tofacitinib (59.02–77.38%) with increasing concentration of mucin (0.1–0.3 g) was noticed, which can be associated with the mucin’s highly viscous and hydrophilic nature that resists the diffusion of the drug from the polymeric matrix. A similar trend was noted in the study conducted by Momoh et al. (2020). In their findings, insulin loading was also enhanced with the increase in mucin concentration (19.5–26.5%) [46].

With the rise in CS content (0.2–0.4 g), an increase in drug loading (55.62–64.69%) was observed due to increased swelling ability of hydrogels with increased chitosan concentration. Increased swelling rate resulted in more interaction between drug molecules and the polymeric network leading to the higher retention of drug within the network. Bai et al. (2018) worked on temperature and pH-sensitive chitosan-based hydrogels. By using a PBS solution, they achieved 98% loading of soluble drug BSA [47].

Formulations containing MAA (8–14 g) exhibited an increasing trend (69.25–80.52%) in drug loading (%). Ionization of carboxylic groups (-COOH) into carboxylate ions at pH 7.4 promotes chain relaxation and swelling resulting in enhanced tofacitinib loading (%). A similar trend was found by Mahmood et al. (2019), where the loading of the drug lovastatin was also promoted with the rise in MAA contents at pH 7.4 [48].

When the concentration of MBA was increased from 0.3 to 0.7 g, drug loading percentage declined (72.53–49.25%). This lowered drug loading capacity is likely related to the increase in crosslinking density of MBA that reduces the elasticity between polymeric chains. Thus, it provides less space and restricts drug entrance into hydrogels. Hence, this results in decreased tofacitinib loading (%). Malik et al. (2020) formulated xanthan gum and chitosan-based hydrogels. In their study at pH 7.4, there was a decrease in drug loading with enhanced MBA content, which is similar to our findings [49].

### 2.8. Equilibrium Swelling Studies (%)

**Figure 8:**
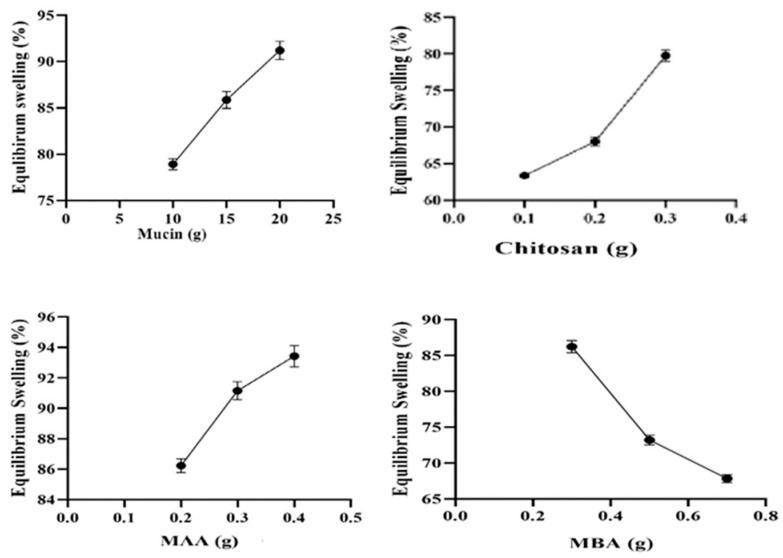
Mucin, chitosan, MAA, MBA effect on equilibrium swelling (%) at 6th h.

Swelling studies are extensively used for preliminary evaluation of hydrogel microparticles in terms of pH sensitivity. It is a key parameter associated with drug loading and release of therapeutic agents from the polymeric network. The study was carried out to examine any impact of the acidic or basic medium on the swelling property of the hydrogel microparticles developed from variable contents of ingredients used, i.e., mucin and chitosan (polymers), MAA (monomer), and MBA (crosslinker). All the prepared formulations (MCT1–MCT12) were evaluated by soaking hydrogel microparticles in buffer solutions of pH 1.2 and pH 7.4, separately. A negligible swelling rate, i.e., less than 15%, was observed in phosphate buffer of pH 1.2 while pronounced swelling was noticed in phosphate buffer of pH 7.4. The results are depicted in Figure 8.

Formulations (MCT1–MCT3) with variable concentrations of mucin (0.1–0.3 g) presented increased swelling (78.21–91.07%). Mucin has hydrophilic moieties together with a well-distributed amino group in its structure. This offers the formation of a more open polymeric network, markedly favoring the absorption of an analyte that can ultimately result in enhanced swelling capacity. Formulations (MCT4–MCT6) containing different amounts of chitosan (0.2–0.4 g) exhibited an increasing trend for equilibrium swelling, i.e., 63.54–79.86%. At basic pH, carboxylic groups attain a negative charge due to the ionization of -COOH and the NH_3_^+^ group converts to a NH_2_ group. Furthermore, the polymer formed no ionic links under these conditions, resulting in decreased crosslinking density. Hence, favoring increased uptake of physiological fluid and enhanced swelling capacity. Surya et al. (2020) have prepared pH-sensitive hydrogels based on chitosan and succinic anhydride, in which swelling was also promoted at pH 7.4 with the increase in chitosan concentration as in our study [50].

Formulations (MCT7–MCT9) containing variable quantities of MAA (8–14 g) showed swelling in increasing fashion, i.e., 86.01–93.62%. At pH 7.4 the carboxylic group of MAA attained ionized state with the release of H^+^ ion leading to charge repulsion and opening up of the spaces between polymeric chains. This allowed for the penetration of swelling media into the polymeric network resulting in increased swelling ability. In a study by Mahmood et al. (2019), there was a rise in swelling at pH 7.4 with MAA as observed in our findings [48].

Formulations (MCT10–MCT12) having different contents of MBA (0.3–0.7 g) displayed swelling percentage in a decreased manner, i.e., 86.17–67.23%. An increase in MBA quantities resulted in the enhanced crosslinking ability of the network that revealed denser and harder structure. Hydrogels with decreased pore size were obtained due to dense structure that led to reduced penetration of swelling media; hence, low swelling was observed. At pH 7.4, studies by Bashir et al. (2020) revealed that there was a decline in swelling percentage from 84.62–48.25% with the increase in MBA concentration [51].

### 2.9. In Vitro Dissolution Studies and Release Kinetics

**Figure 9:**
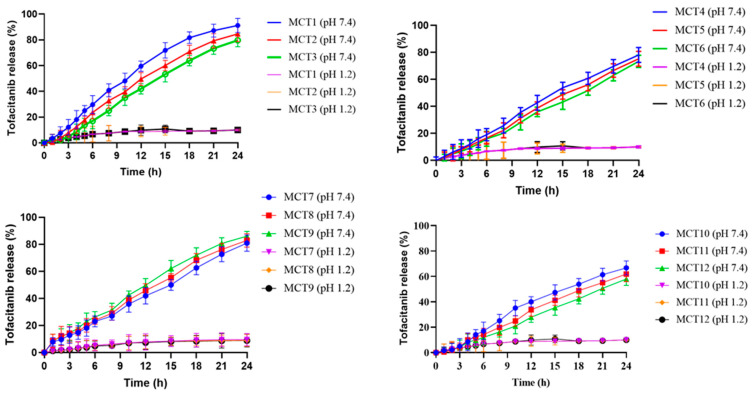
Results of tofacitinib release from developed formulations (MCT1–MCT12).

Release studies for developed MU-CHI-Co-Poly (MAA) hydrogel microparticles were carried out as revealed in Figure 9. The effect of varying concentrations of polymers, monomer, and crosslinker on the release of tofacitinib was analyzed at pH 1.2 and 7.4. Minimum drug release, i.e., less than 20%, was observed at pH 1.2, while there was increased drug release at pH 7.4. 

Drug release (%) was increased (79.88–92.31%) in formulations (MCT1–MCT3). It can be related to the hydrophilic nature of the polymer as well as increased ionizable -OH groups. Ionization between -OH groups results in repulsion, which leads to relaxation and opening of spaces between polymeric chains and enhanced drug release. Mumuni et al. (2020) prepared mucin and PEG-based microparticles with insulin as a model drug to study drug release. Less than 12% drug release was noticed at pH 1.2, while 68–92% insulin release was observed at pH 7.4. Drug release (%) was promoted (76.92–82.63%) in formulations (MCT4–MCT6). The optimum release was obtained for MCT4 (82.63%). For formulation MCT6, the higher chitosan concentration resulted in increased path length, which delayed the release of tofacitinib from microparticles. Bashir et al. (2016) formulated chitosan-based hydrogels. In their study, drug release was increased at pH 7.4 compared to acidic conditions [52].

Drug release (%) was enhanced (78.02–90.56%) in formulations (MCT7–MCT9). The increase in tofacitinib release was related to the ionization of the -COOH group present in MAA. Ionization resulted in polymeric repulsion and expansion. This expansion led to increased swelling, which ultimately increased drug release. Abbasi et al. (2019) prepared pH-sensitive hydrogel using the drug sulfasalazine. In their results, drug release was increased with increased MAA concentrations, which was similar to our results [53]. Drug release (%) was seen to be decreased (57.32–66.43%) in formulations (MCT10–MCT12) containing MBA (0.3–0.7 g). The reason for decreased tofacitinib release may be related to the increased crosslinking, resulting in minimal pore size and a denser polymeric network. Study by Gangadharappa et al. (2017) also concluded that with the increase in concentration of MBA at pH 7.4 there was a decrease in drug release [54].

Kinetic models were employed to determine the best fit model and release mechanism of tofacitinib from hydrogel microparticles. Zero-order, first-order, Higuchi and Korsemeyer–Peppas kinetic models were applied to the release data obtained from all the formulations (MCT1–MCT12). From the R^2^ value, it was confirmed that the best fit model was zero-order. The release of drug at T25, T50, and T75 was 7.72, 15.45, and 23.18, respectively, thereby establishing the controlled release of tofacitinib from the polymeric network. The value of ‘n’ obtained from Korsemeyer–Peppas suggested that the drug followed super case II transport. Results are shown in Table 2.

### 2.10. Sol–Gel Fraction

The analysis aimed to determine the concentration of polymers, monomer, and crosslinker, which remained unreactive during the polymerization reaction. The crosslinked part is ‘gel fraction’ and the portion not crosslinked during the formulation process of hydrogels is known as ‘Sol fraction’.

Formulations (MCT1–MCT3) presented increased gel fraction (82–86%) with increased mucin concentration. Noncovalent bond formation through intermolecular interactions may be the reason for enhanced gel fraction in the polymeric network. Formulations (MCT4–MCT6) showed an increasing trend of gel fraction (94–98%) when the amount of chitosan was increased from 0.2 g to 0.4 g. An increase in gel fraction may result from macromolecules production due to free radicals, which leads to a polymerization reaction. Rehman et al. (2021) worked on chitosan and agarose-based pH-responsive hydrogels for targeted drug delivery of capecitabine. Their findings showed a progressive increase in gel percentage when the amount of chitosan was enhanced [55].

Formulations (MCT7–MCT9) with variable quantities (8–14 g) of MAA exhibited increased gel fraction, i.e., 90% to 96%. The rise in gel fraction may be due to presence of carboxylic groups in the monomer structure. They present a strong affinity for reactive sites on fabricated hydrogel network that facilitate the polymerization reaction between ingredients. These findings were similar to results of Batool et al. (2021) where with the increase in MAA concentration, there was an increase in gel fraction [56].

Formulations (MCT10–MCT12) containing variable quantities (0.3–0.7 g) of MBA demonstrated an increase in gel fraction, i.e., 74% to 86%. An increase in gel fraction with the increase in MBA concentration may be due to the high crosslinking ability of crosslinker at elevated quantities. Sohail et al. (2021) revealed that by increasing the MBA concentration, crosslinking between formulated hydrogels also increased, leading to enhanced gel fraction [57].

**Figure 10:**
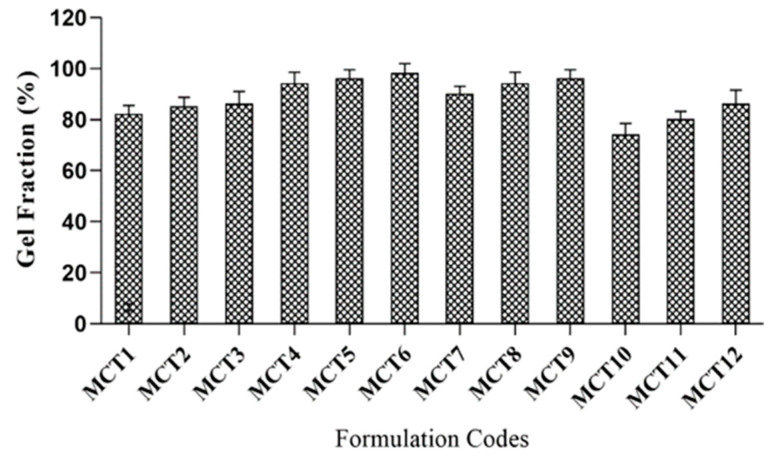
Gel fraction (%) results of developed formulations (MCT1–MCT12).

Results enumerated that sol fraction was reduced while an increase in gel fraction was noticed. All formulations exhibited gel fraction (%) higher than 70%, demonstrating the crosslinked polymeric network’s successful formation. Boiling distilled water was employed as solvent for measurements of sol–gel fraction (%) of developed formulations (MCT1–MCT12). All the results are presented in Figure 10.

### 2.11. Hydrogel Microparticles Size and Zeta Potential Determination

**Figure 11:**
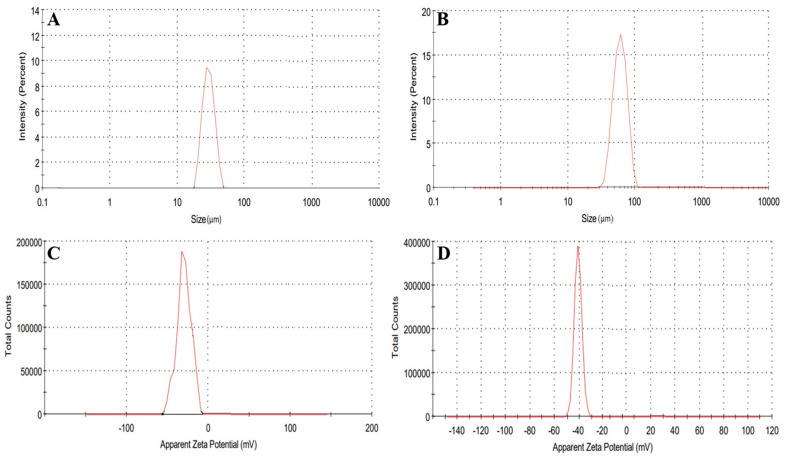
Zeta size of unloaded (**A**) (MCT9) and tofacitinib-loaded hydrogel microparticles (MCT9) (**B**), zeta potential of unloaded (**C**) (MCT9) and loaded MU-CHI-Co-Poly (MAA) hydrogel microparticles (**D**) (MCT9).

Size and zeta potential measurements of the developed MU-CHI-Co-Poly (MAA) hydrogel microparticles were carried out. The size of the formulated polymeric microparticles was in the range of 1–100 µm. Mostly, unloaded microparticles presented a size of 36 µm (Figure 11A). However, loaded microparticles were slightly larger, i.e., 85 µm due to the presence of the drug within the polymeric matrix. Results are exhibited in Figure 11B.

To check the stability of fabricated unloaded and loaded microparticles, zeta potential studies were conducted. An ideal zeta potential value is −40mV. The zeta potential of unloaded and loaded hydrogel microparticles was −30mV (Figure 11C) and −41 mV, respectively. The overall negative charge on the surface leads to repulsion between the developed particles thereby ensuring better stability and providing resistance against aggregation. Results are presented in Figure 11D.

### 2.12. Permeation Studies

**Figure 12:**
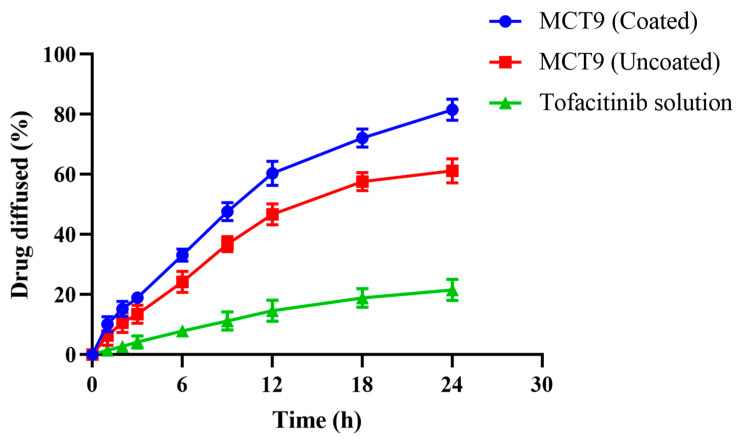
Ex vivo permeability studies in chicken intestine.

MCT9 was selected for permeation studies based on the results of higher swelling, better dissolution and excellent drug loading. A permeability study was conducted at pH 7.4 on coated and uncoated microparticles (MCT9) following the procedure in Section 4.1.2. Tofacitinib offers low permeability in the intestine. Coated microparticles depicted increased drug diffusion (83.44%) as compared to uncoated formulation (65.34%) and pure drug solution (21.56%). Results are presented in Figure 12.

Eudragit (EU) is a copolymer of MMA synthesized from the esters of MAA and approximately has a molar ratio of 2:1. The presence of quaternary amine groups as salts in the EU results in increasing the permeability of the drug. Furthermore, the addition of surfactant, i.e., SLS causes a decrease in surface tension on the membrane surface and the opening of tight junctions. This could lead to the extraction of intracellular lipids and actively prevent efflux, which ultimately enhances the permeation of tofacitinib across the intestine.

### 2.13. Anti-Inflammatory Studies

This study was conducted in seven groups containing four rats each to evaluate the anti-inflammatory effects of the prepared formulations. Group II- and III-administrated standard drug solution presented dose-dependent behavior with a significant (*p* < 0.001) decrease in paw edema at a dose of 10 mg/kg. Group IV presented a significant reduction in inflammation at a dose of 15 mg/kg, whereas Group VI received 17 mg/kg and exhibited a nonsignificant (*p* < 0.05) reduction in inflammation, but after 2 h, decreased paw volume was observed. The anti-inflammatory effects of both test formulations at 30 and 34 mg/kg (Groups V and VII, respectively) was found to be therapeutically significant (*p* < 0.05) after 1h of injecting rats with carrageenan. However, over time, Group V (30 mg/kg) showed an evident decrease (*p* < 0.001) in paw edema in comparison to Group VII (34 mg/kg). All the results were compared with the control group. Table 3 shows the effect of test formulations and standard drug against carrageenan-induced paw edema.

Standard drug after 3 h of induction of rats with carrageenan exhibited pronounced inhibition of 33% at a higher dose, i.e.,10 mg/kg, compared to the low dose of 5 mg/kg. Both formulations A and B presented a dose-dependent decrease in paw volume. The inhibitory effect for both formulations at 15 mg/kg was comparable to the standard drug but showed better results at a dose of 30 mg/kg body weight. Formulation A, at the higher dose (30 mg/kg), produced significant inhibition of 32% in paw edema. Furthermore, in formulation B, at 17 mg/kg dose, 17% inhibition and at 34 mg/kg dose, 33% inhibition was observed after 3h of injecting carrageenan. The inhibition percentage of test compounds is presented in Table 4.

### 2.14. Oral Toxicity Studies

An acute oral toxicity study was conducted to determine the safety level of formulated MU-CHI-Co-Poly (MAA) hydrogel microparticles. The procedure adopted was according to the guidelines provided by the OECD. No mortality or sign of toxicity was observed in treated groups of rabbits after oral administration of microparticles. Moreover, no physical change was seen in control as well as treated group during the 14-day observation period.

#### 2.14.1. Clinical Manifestations

Rabbits under study were physically examined daily to inspect for any sign of illness. Different parameters, i.e., body weight, food intake, heart rate, breathing, water intake, mortality, and dermal and ocular toxicity, were monitored. No noticeable pathophysiological alterations in both groups (treated and control) were noticed during this study period. Results are presented in Table 5.

#### 2.14.2. Blood Analysis

Blood samples were obtained from both rabbit groups on the 1st and 14th day at different time periods to determine CBC, LFT, RFT, and uric acid values. Albino rabbits of treated and control groups were sacrificed on the 14th day to acquire vital organs, i.e., stomach, spleen, brain, kidney, lungs, liver, and heart. Removed organs were dipped in 10% formalin solution and tissue slides were prepared to conduct histopathological examination.

Results attained after hematological (Table 6) and histopathological evaluation were per the standard reference range. Renal, lipid, and liver profiles of treated and control groups are depicted in Table 7. Data presented in Table 6 revealed no significant change in blood profile, confirming that developed MU-CHI-Co-Poly (MAA) hydrogel microparticles were safe, nontoxic, and biocompatible.

#### 2.14.3. Histopathological Examination

**Figure 13:**
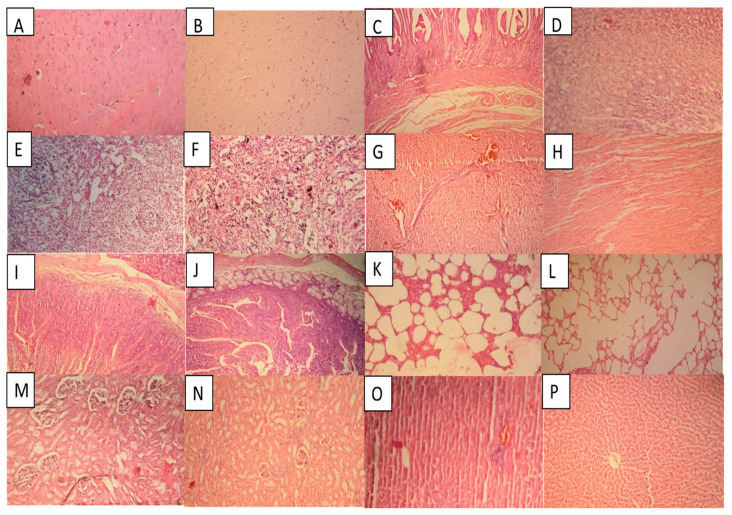
Histopathological examination of (**A**) = brain (control), (**B**) = brain (treated), (**C**) = stomach (control), (**D**) = stomach (treated), (**E**) = spleen (control), (**F**) = spleen (treated), (**G**) = heart (control), (**H**) = heart (treated), (**I**) = intestine (control), (**J**) = intestine (treated), (**K**) = lungs (control), (**L**) = lungs (treated), (**M**) = kidney (control), (**N**) = kidney (treated), (**O**) = liver (control), (**P**) = liver (treated).

Histopathological examination of brain tissues of both treated and controlled groups exhibited normal brain tissues with visible nerve cells wrapped around in myelin sheath. No indication of inflammation or infiltrating of immune cells was observed. Results are depicted in Figure 13A,B. Histopathological examination of stomach tissues of both groups (treated and controlled) showed no signs of inflammation and presented intact stomach linings. There was no indication of gastric ulceration. Results are exhibited in Figure 13C,D. Histopathological examination of the spleen tissues of both groups showed an overall normal appearance without any indication of inflammation. There was not any sign of morphological variation. The normal shape of the spleen section was maintained in both treated and controlled groups. Results are exhibited in Figure 13E,F. Histopathological examination of heart tissues of the treated group showed minute signs of tissue disruption. Regeneration tissues with progressive fibrotic areas were observed. Cardiomyocytes were normal in size, with no indication of hypertrophy.

Moreover, in the control group, there was no sign of cell injury or inflammation. Cells in normal size with no blood clots were seen. Results are shown in Figure 13G,H. Histopathological examination of intestinal tissues of both groups, i.e., treated and controlled showed intestinal epithelium was intact. Lymphatic capillary (lacteal) was present. Moreover, intact serosal layers and smooth muscle tissues were observed. Results are exhibited in Figure 13I,J. Histopathological examination of the lung tissues of the treated and the control group of rabbits showed minor signs of hyperplasia and inflammation. However, pulmonary hemorrhage was not observed. Accumulation of fluid or pulmonary emphysema was also not found. Results are presented in Figure 13K,L. Histopathological examination of kidney tissues of both groups, i.e., treated and controlled, indicated normal glomerulus morphology surrounded by a Bowman’s capsule. Normal structure and shape of renal tubes and no cellular damage was observed. There was no accumulation of inflammatory cells. Furthermore, no signs of hemorrhage or necrosis were found. Results are shown in Figure 13M,N. Histopathological examination of liver tissues (treated and controlled group) displayed no signs of necrosis or degradation in hepatic cells. A classic liver lobule with central veins was observed. Liver sinusoids were devoid of any abnormality, i.e., hypertrophy or active hyperemia. There was no infiltration of lymphocytes, macrophages or neutrophilic granulocytes. Results are presented in Figure 13O,P.

## 3. Conclusions

Novel MU-CHI-Co-poly (MAA) hydrogel microparticles were developed successfully using free-radical polymerization. The developed microparticulate system was tuned for better swelling and release at the desired site. Zeta potential measurements and thermal evaluation ensured the stability profile of developed carrier system. Toxicological evaluation endorsed biocompatibility of the developed carrier system against vital organs. We can conclude that the prepared polymeric system holds great potential for sustained drug delivery and permeability enhancement of the antirheumatic agent tofacitinib.

## 4. Materials

Tofacitinib was received as a generous gift from Brooks Pharmaceuticals, Karachi, Pakistan. N, N methylene bisacrylamide was procured from Fluke, Switzerland. Ammonium persulfate was purchased from AppliChem GmbH, Darmstadt, Germany. Potassium dihydrogen phosphate and sodium lauryl sulfate (SLS) were purchased from Merck, Darmstadt, Germany. Chitosan, Mucin, and Methacrylic acid were purchased from Sigma-Aldrich, Burlington, USA. All the chemicals were used as such as received. Deionized water was freshly prepared in the Postgraduate Research Lab of the Faculty of Pharmacy, The University of Lahore.

### 4.1. Methods

#### 4.1.1. Development of MU-CHI-Co-Poly (MAA) Hydrogel Microparticles

**Figure 14:**
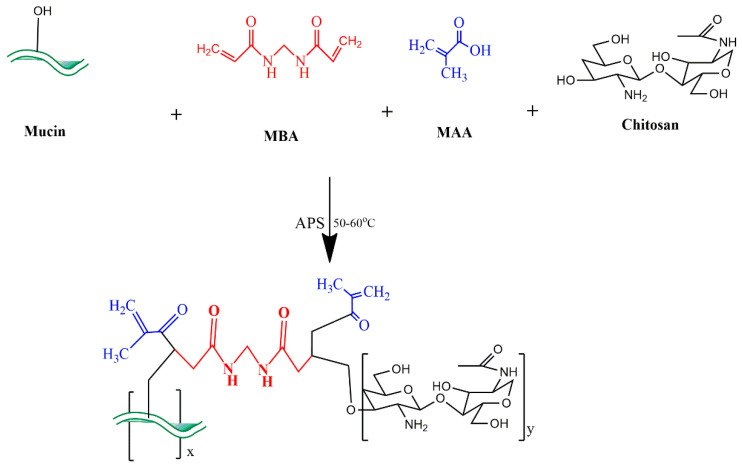
Proposed chemical structure of MU-CHI-Co-poly (MAA) hydrogel microparticles.

Mucin and chitosan based hydrogels were developed using the free radical polymerization method with minute changes. Twelve formulations (MC1–MC12) (Table 8) with various concentrations of each ingredient were developed. Firstly, the weighed amount of mucin was stirred with 7–10 mL of freshly prepared distilled water using a magnetic hotplate stirrer (J-HSD180, JISICO, Seongdong, Korea) at 100 rpm for 5–10 min. The solution was then sonicated for 5 min at 40 °C and centrifuged for 4–5 min at 5000 rpm. The supernatant solution was collected into a beaker. In another beaker, chitosan was dissolved in 7–10 mL of acetic acid (1%) solution and magnetically stirred for 5 min at 100 rpm. A weighed amount of APS was incorporated in 1–2 mL of distilled water to form a separate solution. The prepared mucin and chitosan solutions were mixed together and stirred with a dropwise addition of APS solution to generate active sites. In another beaker, MBA was pre-dissolved in water, followed by MAA. The polymer solution was then added to the monomer solution with magnetic stirring for 5–10 min. The resultant solution was transferred into washed, dried and labeled glass test tubes. These test tubes were sealed with aluminum foil after performing sonication for 5 min at 40 °C. Test tubes containing solution were kept in a water bath using test tube stand at 55 °C for 1h and then at 60–65 °C for the next 2–3 h. After hardening the solution, test tubes were taken out of the water bath, kept at room temperature to cool down. Prepared hydrogel rods were removed from glass test tubes using a micro-spatula and shifted to clean and labeled Petri dishes. Hydrogel rods were then carefully cut down into required/small sizes with a sharp cutter then washed with a mixture of methanol and water (30:70) solution to remove any unreacted substances. After washing, hydrogel discs were removed using a sieve and placed into labeled Petri dishes to be dried in an oven at 40 °C for 2–3 days. Dried hydrogel discs were crushed in a pestle and mortar and sieved through mesh size 40 to acquire hydrogel microparticles. Then, they were placed in airtight containers to be used for further studies [32]. The proposed chemical structure of the newly developed network is shown in Figure 14.

#### 4.1.2. Coating of Hydrogel Microparticles

A reported method with slight changes was adapted to coat hydrogel microparticles. Ideally, four formulations (MCT3, MCT6, MCT9, and MCT12) were chosen because of optimum contents of polymers, monomer (MAA), and crosslinker (MBA), respectively. From each selected formulation, hydrogel microparticles were taken and accurately weighed, and their weight was noted. Coating solution (2%) was then prepared by dissolving Eudragit^®^ RS-100 in dichloromethane. Required quantity (1 g/100 mL) of SLS was also dissolved in this solution. Microparticles were carefully poured into beaker containing coating solution and subjected to stirring for 3 to 5 min. Afterward, coating solution containing microparticles was subjected to filtration and coated microparticles were recovered on filter paper. Coated microparticles were air dried and stored in closed container for further use [33].

### 4.2. Characterization

#### 4.2.1. Elemental Analysis

This study was conducted to evaluate the elemental composition of samples. This technique enables the evaluation of atomic masses and type of elements in the materials under study. The analysis of the elemental composition was conducted for pure drug and drug loaded and unloaded hydrogel microparticles using PentaFET-6900 (Oxford, UK) at a voltage of 20 KV [58,59,60].

#### 4.2.2. Fourier Transform Infrared Spectroscopy

FTIR spectra of tofacitinib, polymers (mucin, chitosan), and different formulations of loaded and unloaded microparticles were recorded. Samples were grinded and placed in the oven overnight for complete dryness. The 2 mg sample was further grounded with 98 mg of KBr. Samples were then further processed in a hydraulic press to form pallets for scanning. Samples were scanned at a range of 4000–400 cm^−1^ to analyze molecular changes and functional groups in the prepared hydrogel microparticles at room temperature of 25 °C [26,51].

#### 4.2.3. Differential Scanning Calorimetry

The heat stability of pure drug, polymers, and loaded and unloaded polymeric formulations were investigated using Shamidzu calorimeter, DSC-60 (Tokyo, Japan). Each sample (4 mg) was placed in an aluminum pan, which was hermetically sealed. All the samples were kept under the nitrogen atmosphere at a flow rate of 50 mL/min and exposed to the heating system at 15 °C/min. Samples were analyzed at a temperature range of 25 °C to 500 °C [26].

#### 4.2.4. Thermogravimetric Analysis

TGA analysis was conducted to evaluate the thermal stability of drug-loaded and unloaded MU-CHI-co-poly (MAA) hydrogel microparticles and all other components used in the formulation using TGA 2 (Mettler, Switzerland). Triturated microparticles were used. A microbalance attached to a platinum open pan with a capacity of 100 μL was used to measure 0.5–5 mg of sample. The temperature was maintained between 25–500 °C with a heating speed of 20 °C min^−1^ in a nitrogen atmosphere. All the measurements were performed in a set of three [61,62].

#### 4.2.5. X-ray Diffraction Analysis

*XRD* studies were carried out in order to the confirm nature, i.e., crystalline or amorphous, of the research drug, an unloaded network and drug-loaded hydrogel microparticles were petri dishes. Samples were scanned over a scanning range of 10–80° using a powder X-ray diffractometer (Bruker Kahlsruhl, Germany) at room temperature [20].

#### 4.2.6. Scanning Electron Microscopy

The morphology of the formulated MU-CHI-Co-Poly (MAA) hydrogel microparticles was examined by using a JEOL JSM-7500F (Japan) with an operating potential range of 1 KV. The photomicrographs were taken from dried, swollen loaded and unloaded microparticles. Samples were subjected to swelling at 25 °C in deionized water for 48 h. Hydrogel microparticles were carefully cut down by a sharp blade and lyophilized at −70 °C. Photomicrographs were taken by randomly scanning dried microparticles by mounting them on gold-coated aluminum stubs with a thickness of around ~300 Å under vacuum and Ar atmosphere [20,61].

#### 4.2.7. Drug Loading (%)

All the prepared formulations were subjected to drug loading using the swelling diffusion method. A drug solution of 1% (*w*/*v*) was prepared by dissolving 1 g of tofacitinib in 100 mL phosphate buffer saline (PBS) solution (pH 7.4). One gram of the dried MU-CHI-Co-Poly (MAA) microparticles were soaked into 1% drug solution until a constant weight was obtained. Microparticles were removed from the solution and washed with distilled water to remove any drug residue on the hydrogel surface. To completely remove the absorbed solvent, drug loaded microparticles were first dried at a room temp of 25 °C and then transferred to an oven for about three days where the temperature was maintained at 40–45 °C for complete drying [63].

Equation (1) was used to measure drug loading is as follows:

(1) $$DL\;\left(\%\right)=\frac{W_{f}-W_{i}}{W_{f}}\times 100\;$$

*W_f_* = Final weight of microparticles after drug loading and *W_i_* = initial weight of empty microparticles before drug loading.

#### 4.2.8. Equilibrium Swelling Studies

The swelling study (teabag test) for all the prepared formulations was conducted in phosphate buffer solutions of pH 1.2 and pH 7.4 at room temperature to evaluate the pH-sensitive behavior of the formulated MU-CHI-Co-Poly (MAA) hydrogel microparticles. The prepared microparticles were dried, crushed, and passed through a sieve of the required size. A weighted amount of 1 g of the microparticles was placed in teabags of the brand Lipton after removing the tea and carefully sealed. Tea bags were then submerged in the buffer solutions of pH 1.2 and 7.4 in separate beakers. Bags were removed from the buffer solutions at predetermined time intervals, i.e., 0, 0.5, 1, 2, 3, 4, 5, and 6 h. A blotting paper was used to clean their surface and then tied on the burette till no liquid dripped from the tea bags. The bags were reweighed after swelling then re-immersed again in the buffer solutions after weighing [64]. The swelling of the polymeric microparticles was calculated using Equation (2):

(2) $$Equilibrium\;Swelling\;\left(\%\right)=\frac{Ws-Wd}{Wd}\times 100$$

*Ws* = weight of polymeric microparticles after swelling

*Wd* = weight of dried polymeric microparticles before swelling

#### 4.2.9. In Vitro Drug Release and Kinetic Modeling

The dissolution study was conducted to observe the potential site of drug release in USP dissolution apparatus II. To analyze the site-specific drug delivery either in SGF or SIF, two different buffer solutions were prepared and used as dissolution media. Buffer solution of pH 1.2 was prepared with 0.2 MKCl and 0.2 MHCl, whereas for pH 7.4, 0.2 M NaOH and 0.2 M KH_2_PO_4_ were used. Dissolution was carried out using 900 mL of each buffer solution at 50 rpm as a speeding rate and temperature was kept at 37 °C. A 5 mL sample was collected from each vessel at zero-time interval to up to 24 h. Aliquot obtained was subjected to UV-spectrophotometric analysis at a wavelength of 275 nm. Same volume of fresh buffer solution was added each time after withdrawal to maintain the constant volume throughout the release studies [65].

Data obtained from release studies were analyzed by applying kinetic models (Equations (3)–(6)) with the usage of DD solver, an add-in program of Excel. The drug release mechanism followed by the formulated hydrogel microparticles was confirmed based on the R^2^ value (best fit model) and from ‘*n*’ value. If the value of ‘*n*’ is equal to 0.45, then it is Fickian diffusion, but follows non-Fickian diffusion when the value of ‘n’ is greater than 0.45 but less than 0.89. If the value of *n* > 0.89, then it is case II/zero order.

Zero-order kinetics

(3) $$Q_{t}=Q_{o}-K_{o}t$$

First-order kinetics

(4) $$L_{n}Q_{t}=L_{n}Q_{o}-K_{1}t$$

Higuchi model

(5) $$Qt=K_{t}H^{\frac{1}{2}}$$

Korsemeyer–Peppas model

(6) $$\frac{M_{t}}{M_{\infty }}=Kt^{n\;}$$

*Qo* is the amount of drug released from hydrogels at time *t* = 0, *Qt* and *Mt*/*M*∞ are the release of drug at time ‘*t*’. *n* is the release exponent used to determine drug release mechanism, whereas *K*, *K_H_*, *k*_1_, and *K_o_* are the release rate constants [66].

#### 4.2.10. Sol–Gel Fraction

This study was conducted to determine the number of reactants utilized during the development of the polymeric network. For this evaluation, known quantities of all the prepared microparticles were taken and triturated with pestle and mortar into small sizes of about 2 mm. Soxhlet apparatus was used for the extraction. Analysis was carried out for 4 h with the temperature being set at 95 ± 5 °C. Recondensation was performed in a round-bottom flask filled with boiling distilled water for soluble reactants. Hydrogel microparticles were then taken out and dried at 20–22 °C and finally placed in the oven for drying at a temperature range of 40–45 °C [60]. For calculating the sol fraction, Equation (7) was used:

(7) $$\mathrm{Sol}\;\mathrm{fraction}\;\left(\%\right)=\frac{W_{f}-W_{i}}{W_{f}}\times 100$$

Gel fraction (%) = 100—Sol fraction.

*W_i_* = initial weight of dried hydrogel microparticles and *W_f_* = final weight after extraction process.

#### 4.2.11. Hydrogel Microparticles Size and Zeta Potential Determination

Size and zeta potential measurements were performed based on dynamic light scattering technique diffraction using zeta sizer (Zetasizer Nano ZS, Malvern, Instruments, UK) for developed loaded and unloaded MU-CHI-Co-Poly (MAA) hydrogel microparticles (MCT9). For this analysis, formulated microparticles were dispersed in distilled water. Quasi-elastic light scattering was used for the observations. Stability and ionization were studied with zeta potential. Particles having an electric potential value of less than −40 mV are repulsive to each other, thus ensuring the stability of the prepared hydrogel microparticles [67,68].

#### 4.2.12. Permeation Studies in Chicken Intestine

The intestine of a broiler chicken was used to conduct permeation analysis. The intestinal segment was obtained from the chicken center near University of Lahore (Lahore) on the same day of the experiment and used within 2 h. The intestine was cut down into pieces 6 cm in length with a sharp blade and turned inside out by rolling the sliced-out part on a glass rod. Normal saline (0.9% NaCl) was used to wash the isolated parts of the intestine. One end of the intestine was tied up with a thread forming a sac filled with a buffer solution of pH 7.4 (25 mL) as a release media for tofacitinib. Afterward, sac was hung onto the dissolution apparatus and the selected formulated microparticles (MCT9) were placed in the basket. Permeation studies were carried out at 37 ± 2 °C and a rotating speed of 50 rpm. Two mL sample was taken out from the sac and analyzed after filtration (syringe filtration assembly containing 0.2µm pore size Sartorius filter paper) at 275 nm using UV spectrophotometer. All the results were calculated with the repetition of three [69].

#### 4.2.13. Anti-Inflammatory Activity

MU-CHI-Co-Poly (MAA) microparticles (MCT9) were evaluated for anti-inflammatory potentials, where inflammation was first induced with carrageenan in the paw of the rat. After 24 h of fasting with free access to water, the animals, weighing 150–200 g, were categorized into 7 groups with four rats in every group. The control group (Group I) was served with 2 mL/kg distilled water. Groups II and III were administered 5 and 10 mg/kg of tofacitinib solution, respectively, and were considered as standard. Moreover, Groups IV, V, VI, and VII were provided the prepared microparticles P.O. in doses of 15, 30, 17, and 34 mg/kg, respectively. After 1 h of the treatment, acute inflammation was induced with an injection of freshly prepared 0.1 mL of carrageenan solution (1%) in the left hind paw of rat. The injection site was marked, and readings were taken in ml with a plethysmometer. The diameter of injected left paw was measured at 0, 1, 2, and 3 h intervals. The differences in the volume of the paw measured initially and after the induction of inflammation at different time intervals. The percentage inhibition for the standard drug and different doses of developed formulations provided at specific intervals were computed by comparing with the values of control group by using Equation (8):

(8) $$\;Percentage\;inhibition=\frac{{\left(W_{t}-W_{o}\right)}_{control}-{\left(W_{t}-W_{o}\right)}_{treated}}{{\left(W_{t}-W_{o}\right)}_{control}}\times 100$$

where, *W_t_* is the volume of the paw at time ‘*t*’, *W_o_* is the initial volume of paw at time ‘0′. (*W_t_* − *W_o_*)*_control_* are the values of edema in the control group as well as (*W_t_* − *W_o_*)*_treated_* are the values obtained from inflammation in the treated group [70,71].

### 4.3. Acute Oral Studies

#### 4.3.1. Animals

Toxicity and anti-inflammatory studies were reviewed and approved by the Institutional Research Ethics Committee (IREC) (IREC) with approval number IREC-2021-08. Guidelines provided by the OECD were strictly followed while handling animals. All the regulations provided by IREC are the protocols specified by ICH GCP guidelines approved by the FDA to conduct experimental studies in animals [48].

#### 4.3.2. Oral Toxicity Studies

Six rabbits weighing 1.5–2 kg were obtained from the local animal market in Lahore, Pakistan. All the rabbits were kept in the animal house of UOL for one week to be adapted to the environment. Constant temperature ranging 22–25 °C was maintained and animals were provided free access to food and water. To avoid any food and drug interactions, rabbits were in fasting conditions with an excess supply of water before the start of the experiment [35]. Six healthy rabbits were divided into two groups (n = 3). Animals in group A were considered as control while group B was the treated group and was administered powdered MU-CHI-Co-Poly (MAA) hydrogel microparticles (MCT9) at a dose of 2 g/kg. All the animals were carefully observed for any change in body weight, food and water uptake, and skin sensitivity. On the 7th day, 2–3 mL of blood samples were collected in EDTA tubes from the marginal vein of the rabbit ear with a 3cc syringe. Collected samples containing EDTA tubes were shaken by hand so anticoagulant could properly mix with blood. Before analysis, blood samples were centrifuged for 15 min with a rotational speed of 5000 rpm. Anesthesia, i.e., xylazine and ketamine HCl (30:70) (1 mL/kg) was administered to animals on the 14th day. Blood samples from heart were recollected and animals were reweighed and then sacrificed. Vital organs such as heart, lungs, stomach, liver, spleen, brain, kidney, and intestine were removed. Labelled containers containing formalin solution (10%) were used to preserve washed organs and for further histopathological examination [72].

## Figures and Tables

**Table 1:** Elemental composition of tofacitinib, loaded, and unloaded formulations.

Material Type	Elements	% Weight	% Atomic
Tofacitinib	C N O	41.25% 33.72% 25.03%	45.38% 30.23% 24.19%
Unloaded formulation	C O	59.42% 39.15%	70.72% 28.10%
Tofacitinib loaded formulation	O C N	39.24% 34.63% 12.42%	41.02% 30.71% 11.62%

**Table 2:** Results of kinetic modeling of release data (MCT1–MCT12).

Kinetic Models	Parameters	MCT1–MCT12 (Mean)
Zero-order	R^2^	0.998
	T_25_	7.729
	T_50_	15.458
	T_75_	23.186
First-order	R^2^	0.959
	T_25_	6.262
	T_50_	15.089
	T_75_	30.178
Higuchi	R^2^	0.948
	T_25_	4.345
	T_50_	17.380
	T_75_	39.105
Korsemeyer–Peppas	R^2^	0.999
	n	0.993

T25 (Time required for 25% of drug to release from carrier), T50 (Time required for 50% of drug to release from carrier), T75 (Time required for 75% of drug to release from carrier).

**Table 3:** Anti-inflammatory effects of test formulations in carrageenan-induced paw edema.

Group (Treatment/Dose)	Inflammation			
	0 h	1 h	2 h	3 h
Group I (control)	2.07 ± 0.03	2.11 ± 0.05	2.16 ± 0.04	2.21 ± 0.03 ^#^
Group II (5 mg/kg drug solution)	1.86 ± 0.03	1.85 ± 0.04 *	1.81 ± 0.05 **	1.71 ± 0.06 ***
Group III (10 mg/kg drug solution)	2.25 ± 0.03	1.81 ± 0.06 **	1.70 ± 0.07 **	1.49 ± 0.07 ***
Group IV (15 mg/kg microparticles)	1.98 ± 0.04	1.95 ± 0.04 *	1.83 ± 0.03 *	1.80 ± 0.03 **
Group V (30 mg/kg microparticles)	1.96 ± 0.06	1.84 ± 0.04 *	1.71 ± 0.06 **	1.49 ± 0.06 ***
Group VI (17 mg/kg microparticles)	1.95 ± 0.09	1.93 ± 0.05 ^ns^	1.89 ± 0.07 *	1.83 ±0.07 *
Group VII (34 mg/kg microparticles)	1.86 ± 0.08	1.82 ± 0.07 *	1.64 ± 0.07 *	1.48 ± 0.05 **

Data are presented as means of ± SEM by using one way ANOVA. Significant at ^#^ = (*p* > 0.05), significant at * = *p* < 0.05, ** = *p* < 0.01, *** =*p* < 0.001

**Table 4:** Percentage (%) inhibition of carrageenan-induced paw edema in rats at 1 h, 2 h, and 3 h.

Group (Treatment/Dose)	Inhibition (%)		
	At 1 h	At 2 h	At 3 h
Group II (5 mg/kg drug solution)	12 %	16%	23%
Group III (10 mg/kg drug solution)	14%	21%	33 %
Group IV (15 mg/kg microparticles)	8%	15%	19%
Group V (30 mg/kg microparticles)	13%	21%	32%
Group VI (17 mg/kg microparticles)	9%	13%	17 %
Group VII (34 mg/kg microparticles)	14%	22%	33%

**Table 5:** Clinical findings during acute oral toxicity studies.

Observation		Group I (Control)	Group II (Test)
Sign of Illness		NIL	NIL
**Body weight(kg)**	Pretreatment	2.09 ± 2.1	2.15 ± 1.7
	Day 1	2.10 ± 2.0	2.17 ± 1.9
	Day 7	2.12 ± 2.3	2.18 ± 2.2
	Day 14	2.14 ± 2.4	2.21 ± 2.4
**Water intake (mL)**	Pretreatment	177.35 ± 1.31	191.21 ± 0.06
	Day 1	191.65 ± 2.11	192.32 ± 1.51
	Day 7	211.15 ± 2.15	193.21 ± 1.41
	Day 14	216.52 ± 2.19	219.45 ± 2.22
**Food intake (g)**	Pretreatment	76.13 ± 1.33	77.43 ± 1.15
	Day 1	77.33 ± 1.11	77.33 ± 1.17
	Day 7	74.54 ± 1.27	76.55 ± 1.24
	Day 14	77.33 ± 1.18	77.62 ± 1.11
**Dermal toxicity**		Nil	Nil
**Ocular toxicity**		Nil	Nil
**Mortality**		Nil	Nil

**Table 6:** Results of biochemical analysis of rabbit’s blood.

Parameters	Group A (Control)	Group B (Treated)
Hb (10–15 g/dL)	11.19 ± 0.33	12.14 ± 0.33
pH	6.33 ± 0.11	6.43 ± 0.13
WBCs (×10^9^/L)	11.19 ± 0.33	11.41 ± 0.66
RBCs (×10^6^/mm^3^)	5.13 ± 0.144	5.17 ± 0.24
Platelets (×10^9^/L)	259 ± 2.01	262 ± 0.16
Monocytes (%)	3.43 ± 0.055	3.42 ± 0.032
Neutrophils (%)	24.13 ± 2.45	25.24 ± 0.22
Lymphocytes (%)	52.19 ± 1.02	57.12 ± 0.55
MCV (%)	68.55 ± 3.14	66.3 ± 0.22
MCH (pg/cell)	25.13 ± 2.19	24.56 ± 0.08
MCHC (Hb/cell)	29.65 ± 1.88	31.23 ± 0.15

**Table 7:** Liver, renal, and lipid profiles after administration of hydrogel microparticles.

Biochemical Analysis	Group A (Control)	Group B (Treated)
ALT (IU/L)	64.33 ± 4.62	68.50 ± 3.27
AST (IU/L)	72.42 ± 1.55	74.55 ± 1.65
Creatinine (0.8–1.8 mg/dL)	1.5 ± 0.19	1.65 ± 0.31
Urea (mmol/L)	60.30 ± 1.90	61.29 ± 1.21
Uric acid (mg/dL)	3.12 ± 0.14	2.65 ± 0.13
Cholesterol (10–80 mg/dL)	74 ± 1.33	76 ± 1.23
Triglycerides (46–68 mg/dL)	48 ± 1.55	50 ± 1.65

**Table 8:** Composition of MU-CHI-Co-Poly (MAA) hydrogel microparticles (MCT1–MCT12).

Formulation Codes	Mucin (g)	Chitosan (g)	MAA (g)	MBA (g)	APS (g)
MCT1	0.1	0.1	5	0.2	0.1
MCT2	0.2	0.1	5	0.2	0.1
MCT3	0.3	0.1	5	0.2	0.1
MCT4	0.2	0.2	5	0.2	0.1
MCT5	0.2	0.3	5	0.2	0.1
MCT6	0.2	0.4	5	0.2	0.1
MCT7	0.2	0.2	8	0.2	0.1
MCT8	0.2	0.2	11	0.2	0.1
MCT9	0.2	0.2	14	0.2	0.1
MCT10	0.2	0.2	10	0.3	0.1
MCT11	0.2	0.2	10	0.5	0.1
MCT12	0.2	0.2	10	0.7	0.1

## Data Availability

All data are reported in the manuscript.

## Supplementary Materials

The following supporting information can be downloaded at: https://www.mdpi.com/article/10.3390/gels9030187/s1, Table S1: Results of 10% weight loss and Char yield at 500 °C.
