# Supplementary material for: Development of Tofacitinib Loaded pH-Responsive Chitosan/Mucin Based Hydrogel Microparticles: In-Vitro Characterization and Toxicological Screening
Source: Gels. 2023 Feb 28;9(3):187. doi: 10.3390/gels9030187 (PMC10048094; doi:10.3390/gels9030187)
Supplement: Supplementary file 1 [file gels-09-00187-s001.zip › gels-2234183-supplementary.pdf]

Table S1: Results of 10% weight loss and Char yield at 500 °C

Sample	Temperature (◦C) at t = 0	Time for desired temperature (min)	Char Yield*	Temperature (◦C) /time for 10% mass loss (min)
B	27.15	32.19	7.33%	258.19/ 7.14
C	27.12	32.28	32.14%	76.83/ 3.99
D	26.67	32.29	33.44%	198.23/ 12.31
E	27.07	32.53	11.47%	171.88/ 10.7
F	26.9	32.51	45.85%	113.3s/ 6.74

* Char Yield = [(Wf)/Wi]*100
Where Wi equal lto 9 mg for all samples
